# Birth, School, Work, Death, and Resurrection: The Life Stages and Dynamics of Transposable Element Proliferation

**DOI:** 10.3390/genes10050336

**Published:** 2019-05-03

**Authors:** Justin P. Blumenstiel

**Affiliations:** Department of Ecology and Evolutionary Biology, University of Kansas, Lawrence, KS 66049, USA; jblumens@ku.edu

**Keywords:** transposable element, horizontal transfer, arms race, LINE-1, *Alu*, *hobo*, *I* element

## Abstract

Transposable elements (TEs) can be maintained in sexually reproducing species even if they are harmful. However, the evolutionary strategies that TEs employ during proliferation can modulate their impact. In this review, I outline the different life stages of a TE lineage, from birth to proliferation to extinction. Through their interactions with the host, TEs can exploit diverse strategies that range from long-term coexistence to recurrent movement across species boundaries by horizontal transfer. TEs can also engage in a poorly understood phenomenon of TE resurrection, where TE lineages can apparently go extinct, only to proliferate again. By determining how this is possible, we may obtain new insights into the evolutionary dynamics of TEs and how they shape the genomes of their hosts.

## 1. Introduction

“And he that was dead came forth, bound hand and foot with graveclothes.”

John 11:44

Transposable elements (TEs) have an intimate relationship with the genomes of their hosts. Like any form of parasite they cause harm but they are also dependent on the host for fitness. However, unlike typical parasites, they are directly embedded in the genomes of their hosts. How can such parasites spread if they are harmful? Alleles that are harmful are expected to be lost, but transposable elements exist in essentially all forms of life. In eukaryotes, the persistence of TEs is explained by the fact that sexual reproduction allows TEs to spread even if their net effect is a reduction in host fitness. Gamete fusion allows TEs to colonize new genomes [1] and recombination breaks up the association between progenitor copies and harmful descendant copies [2,3]. However, if TEs proliferate too rapidly within genomes, the consequences of their harm can indeed become too high and impede their success [4]. Transposable elements must walk a fine line between a sufficient rate of proliferation and one that is not so great that TEs become too burdened by the harmful effects that they impose.

The nature of this tension depends on the degree of intimacy with the host genome and is illuminated by considering the moment when a TE and the host genome first meet. This occurs during horizontal transfer, which is the first stage in the life cycle of a TE (see reviews on TE life cycles [5,6,7,8,9]). When a TE first invades a genome, it is a particularly fragile moment for the TE family because such events are likely to be serendipitous. For an element to be successful during the early stages of invasion, it must exploit these chance moments and avoid being lost from the population by drift. Studies show that the optimal TE strategy during horizontal transfer is to have a very high initial transposition rate [4,10]. This arises from the fact that the probability that a new TE becomes established is similar to the probability of fixation for a new beneficial allele. In the case of a new beneficial allele, the probability of fixation is ~2*s*, where *s* is the beneficial selection coefficient. For a transposon, the probability of establishment is ~2(*u* − *s*), where *u* is the transposition rate and *s* is the selection coefficient that measures the average harmful effect of each new single insertion [4,10,11]. Establishment is achieved when, on average, each individual in the population has one copy. Rather than fixation, I consider establishment to be a more appropriate term for TE families because fixation is a term that is more appropriate for alleles. Transposable elements insertions within the population are non-allelic if they reside at different locations in the genome. So, if each individual on average carries one insertion, the TE family can be considered established. A single TE insertion allele can be considered fixed if there are no non-insertion variants segregating in the population at that locus.

For both a new beneficial allele and a new transposable element, the fixation (or establishment) probabilities do not depend much on the population size since the dynamics of stochastic loss by drift when the novel variant first appears are the same whether the population size is one million or one trillion. However, a transposition rate that is too high, while it will increase the probability that a TE becomes established, may also impose such a burden that the host may become extinct if the selection regime fails to limit the ever-increasing copy number. Thus, it has been shown that the optimal strategy for a transposable element is to have a high transposition rate during early invasion, followed afterwards by a period with a lower rate of movement [4]. This lower rate of movement may be enabled by host TE suppression mechanisms such as small RNA silencing.

It is not apparent that selection on TE lineages would be efficient enough to directly select such a tunable strategy. However, this tension reveals that optimal TE strategies will depend on the nature of the relationship with a genome. On one end of the continuum, TEs may be long term residents. On the other end, TEs may adopt a strategy of rapid invasion and movement from species to species. In the first part of this review, I discuss the nature and implications of these two strategies. Then, I consider an interesting phenomenon of TE lineages that appear to reside within genomes, go extinct, and then apparently come “back to life” many generations later. I will argue that TEs that show this pattern—I will designate them Lazarus elements—may highlight interesting aspects of TE biology and host interaction.

## 2. Long-Lasting Relationships

Some TE lineages are long-lived residents of their host genomes. In some cases, this is because TEs have adopted a cooperative strategy with the host. For example, in *Drosophila*, telomere function has been assumed by TEs [12]. However, for TEs that remain parasitic with respect to the host, there may be no better example of long-term coexistence than the LINE-1 elements of mammalian genomes. LINE-1 elements are a member of the non-LTR retrotransposon class and have been residents of mammalian genomes since early in the radiation of mammals [13,14,15]. In humans, the LINE-1 element has had a profound role in shaping the genome and there are approximately 500,000 copies of this element [16,17]. The LINE-1 family is shared across most mammals due to continued vertical transmission since early in the mammalian radiation [18,19]. Vertebrates that include reptiles, amphibians and fish also share LINE-1 elements, suggesting that the LINE-1 element may have been present since before the origin of mammals [14]. Alternately, it has been proposed that LINE-1 elements entered the therian mammal ancestor (rather than the ancestor of all mammals) through horizontal transfer. This is suggested by the observation that monotremes lack LINE-1 elements and have no clear signature of their previous activity [20]. In either case, the LINE-1 lineage shows a striking level of persistence and success across mammals through ongoing vertical transmission.

What has enabled this intimate relationship for millions of years within mammals? Phylogenetic analysis of LINE-1 elements within mammals has revealed a particular feature of LINE-1 persistence. In particular, phylogenetic trees of LINE-1 elements within a genome frequently have a “ladder-like” appearance [14,21,22]. This represents a scenario in which, through evolutionary time, there is typically only one or few proliferating lineages. This phylogenetic pattern has been proposed to be driven by an ongoing evolutionary arms-race with the host [14]. In particular, as mechanisms of LINE-1 control evolve on the part of the host, evolutionary innovation on the part of the TE lineage enables escape from host control. Recurrent cycles of adaptation and innovation—in both host and TE—can thus lead to the persistence of a single successful TE lineage [21]. This pattern may also be driven by the smaller effective population sizes that are likely more common in mammals. In very large populations, the fixation of an active and harmful TE insertion allele by drift is unlikely. However, in smaller populations, drift may allow such insertion alleles to fix. When an active copy becomes fixed at a particular locus, only decay into a non-functional state will allow the active copy to be lost from the population. Thus, fixation of an active TE insertion allele represents a critical stage in TE-host dynamics.

Faced with the continued presence of the LINE-1 element over millions of years, specialized modes of host control are proposed to contribute to the evolutionary dynamics that yield the arms-race driven “ladder” phylogeny. In particular, new active LINE-1 lineages may carry key innovations that enable specialized modes of escape from repression [23]. Diverse proteins that restrict LINE-1 transposition include APOBEC3, MOV10, ZAP, SAMHD1 and ZNF93 [24]. Signatures of recurrent LINE-1 adaptation that allow evasion from these restricting factors have also been found. For example, within mammals, the 5’ UTR of LINE-1 is highly dynamic [22,25,26,27]. This has been proposed to be driven by the ongoing evolution of KRAB zinc fingers that can evolve specificity to target particular sequences in LINE-1 for repression. In response to this, it appears that selection on the LINE-1 lineage has driven removal of particular target sequences from the 5’ UTR [28].

The ongoing persistence of one or few evolving LINE-1 lineages is likely enforced by within lineage competition. Otherwise, we might expect different modes of adaptation to evolve on distinct TE lineages, followed by successful diversification. Competition for host factors required for transposition has been proposed to contribute to this dynamic [29]. Strikingly, and in contrast to mammalian systems, the proliferation of one or few LINE-1 element lineages does not seem to apply in other vertebrates [14]. Rather, multiple lineages of LINE-1 elements have expanded and proliferated in the genomes of reptiles, amphibians and fish [30,31,32]. This represents a distinct mode of long-term coexistence within the genomes of non-mammalian species. Differences in demographic history and the strength of selection are likely to contribute to this difference. Compared to mammals, some non-mammalian species with greater LINE-1 diversity also show a stronger signature of selection acting to limit the fixation of TE insertion alleles [33]. This suggests that different selection regimes may contribute to the difference in LINE-1 dynamics between mammalian and non-mammalian species (but see [34]). One difference may arise from differences in the probability of ectopic recombination between dispersed repeats [29]. Selection against ectopic recombination is an important determinant of TE dynamics and a low rate across mammalian genomes may decrease the strength of selection against insertions and allow the accumulation of repetitious sequences [35,36,37,38,39,40]. In addition, if lower levels of ectopic recombination allow greater TE accumulation, persisting copies that fix by drift may intensify competition for host factors. Thus, as genomic copy number increases due to reduced levels of genome-wide ectopic recombination, the magnitude of competition for host factors may increase among competing copies and lineages. This may lead to a greater tendency for a single lineage to outcompete all other lineages. For these reasons, selection on LINE-1 lineages may not simply be to evade host restriction factors. Selection to increase access to host factors that enable transposition, amidst a genome filled with many other copies, may also be critical.

## 3. Horizontal Transfer: Fast, Cheap and Out of Control

“Based on our experience in building ground based mobile robots (legged and wheeled), we argue here for fast, cheap missions using large numbers of mass produced simple autonomous robots...”

Brooks and Flynn. 1989. Fast, Cheap and Out of Control: A robot invasion of the solar system

These contrasting modes of LINE-1 evolution—the proliferation of a few lineages in mammals vs. diversification in reptiles, amphibians and fish—represent two forms of long-term co-existence. As previously indicated, long-term co-existence can also be maintained if TEs adapt a strategy of cooperation, as seen in the case of *Drosophila* telomeres. However, for selfish TEs that display parasitic behavior with respect to the host, another strategy relies on horizontal transfer and recurrent invasion. If TEs have the capacity to invade genomes through horizontal transfer, long-term persistence may be enabled by a ‘live fast, die young’ strategy [41,42]. If a TE family can invade a species, proliferate, and jump to a new species, it may conceivably persist even if it is unlikely to endure within any single species. Studies of the DNA transposon *mariner* in *Drosophila* illustrate how such a strategy is possible [43,44,45]. *mariner* was discovered in *D. mauritiana*, a close relative of *D. melanogaster*. However, its presence within the *D. melanogaster* species subgroup is considered “spotty” [46,47]. In particular, it appears in several close relatives of *D. melanogaster* but is absent from *D. melanogaster* itself. It has apparently been lost. Interestingly, an additional *mariner* lineage is also found in the genomes of other members of the melanogaster species subgroup, including *D. erecta*, but was apparently lost from the *D. melanogaster/D. simulans* clade [48]. This latter *mariner* family also shares 97% sequence similarity with a *mariner* element found in the cat flea, indicating horizontal transfer several million years ago. Overall, these patterns indicate that *mariner* dynamics can be explained by a dynamic process of recurrent horizontal transfer and extinction [48]. In contrast to mammals, it appears that horizontal transfer is rampant in insect species. In a comprehensive analysis of the genomes of nearly 200 insect species, more than 2000 horizontal transfer events were found to have occurred within a span of about 10 million years [49]. Strikingly, the *Tc1/mariner* class of DNA transposons shows the greatest frequency of horizontal transfer. This high propensity for horizontal transfer has been attributed to a lack of dependence on host factors for transposition [50]. *Tc1/mariner cis* regulatory sequences that drive transcription in diverse genomes may also facilitate efficient movement across species [51]. Within a single species, a TE lineage can proliferate if its transposition rate is sufficiently high so that it can increase at a rate faster than its removal due to negative selection. The same principle should also apply across species. If a TE can invade, by horizontal transfer, the genomes of new species at a rate faster than the within species extinction rate, the lineage will also find success. In this case, since TE success depends on being able to move across species, it is unlikely that natural selection will be sufficient for adaptation, on the part of a TE lineage, to a particular host genome. Rather, natural selection will favor a “generalist” strategy that enables movement in the genomes of many species.

## 4. Extinction

Whether a TE is adapted for continued vertical transmission (as observed for LINE-1 elements) or ongoing movement across species (as perhaps observed for *mariner* elements), TE lineages are not guaranteed perpetual success. Rather, they can also go extinct within a species. Across mammals, LINE-1 extinction has been observed in the rhinoceros and lineages of rodents, bats, insectivores and Afrotherians [15,52,53,54,55]. Several mechanisms have been proposed to contribute to LINE-1 extinction. In one scenario, mechanisms of host suppression may be sufficient. It has been noted that the fate of a TE lineage depends on the balance between transposition and the rate of accumulation for degenerating mutations [56]. If the transposition rate is lower than the rate of mutation that renders an element inactive, then the TE lineage will decay. Thus, host control mechanisms that drive a significantly low transposition rate may also drive extinction by decay.

Other factors are also likely to contribute to extinction. TE families may drive other TE families to extinction through direct competition for host factors. For example, LINE-1 extinction in a group of sigmodontine rodents may have been influenced by competition for host factors with an expanding endogenous retrovirus lineage [57,58]. Extinction may also be driven by other TE lineages through direct sequestration of TE-encoded factors that enable transposition. SINE elements, such as the *Alu* element, hijack LINE-1 encoded factors to favor their own increase [59]. Thus, *Alu* amplification may drive LINE-1 extinction through competitive saturation of LINE-1 encoded factors required for LINE-1 transposition [60]. Finally, extinction may also be enabled by a form of lineage “suicide”. In the case of DNA transposons, internally deleted copies may titrate functional transposase from fully functional copies [61]. As internally deleted copies within the genome increase, active DNA transposon lineages may lose sufficient access to their own encoded factors.

**Figure 1:**
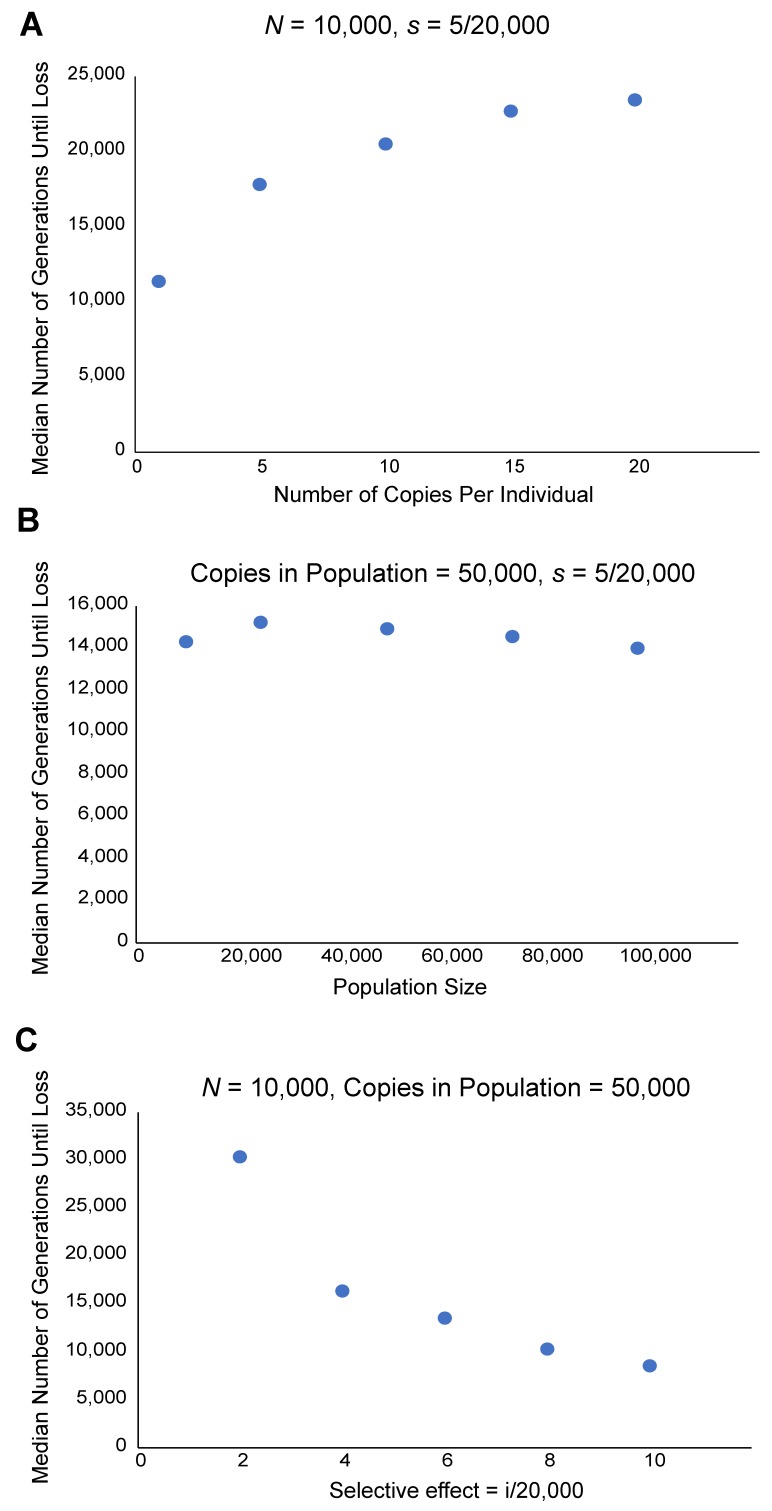
Dynamics of stochastic loss. *N* is the diploid population size and *s* is the selection coefficient acting against single insertions. All simulations were performed by simple binomial sampling of insertion alleles starting at frequency of 1/2*N*. Sampling was iterated according to frequency in the population for a given number of copies. This procedure implicitly assumes there is no linkage. In addition, by assuming no actual transposition or degradation specifically, it is suitable to a scenario where the rate of transposition is equal to the rate of mutation to a non-functional state. Selection was simulated by adjusting the probability of sampling according to the selection coefficient. (**A**) Fixed population size and negative selection coefficient. The time until loss increases with per individual copy number. Note that the rate of increase declines. (**B**) A fixed number of copies in the population, distributed among individuals of different population sizes. The time until loss is not affected by population size. (**C**) An increasing selection coefficient, as expected, decreases the time until loss.

Finally, stochastic loss and demographic factors may also contribute to lineage extinction. In populations where an active TE does not fix at any particular location, selection or drift may simply lead to the loss of every active copy in the genome. This will be most likely when the transposition rate is sufficiently low, so it is likely to be enhanced by host suppression mechanisms. The dynamics of stochastic loss, in many ways, are likely to be similar to loss by the mutational degeneration of active copies. How long will it take for a TE family to be lost by this mechanism? Using simulation, I have shown that total copy number within the population—rather than population size or per genome copy number—dominates the dynamics of stochastic loss assuming no individual insertion becomes fixed (Figure 1). Selection also plays a role.

## 5. Resurrection

Overall, the canonical life-cycle of a TE family starts with invasion followed by proliferation and eventual extinction. The duration for each of these stages may vary and be influenced by a wide variety of factors, as outlined previously. Extinction is certainly not guaranteed but there are many examples of where this appears to be the case. More striking, however, is that in some cases, extinction seems to be followed by resurrection. This is a mysterious phase of TE dynamics and worthy of investigation because it may shed light on the evolution of TE life-strategies that range between recurrent invasion and long-term coevolution.

Resurrection, also known as re-invasion, occurs when an active TE lineage becomes quiescent and perhaps even extinct, and then later proliferates. Syndromes of hybrid dysgenesis were the first to reveal this phenomenon, in particular the *I-R* syndrome of dysgenesis. Hybrid dysgenesis is a syndrome of intraspecific sterility that occurs when active TE families transmitted paternally are absent or nearly absent from the maternal genome [62,63,64]. In the absence of abundant maternal copies, a pool of piRNAs that maintain TE repression is not provisioned to the zygote [65,66]. This leads to activation of paternally inherited TEs and sterility. Perhaps the most well understood syndrome of hybrid dysgenesis is the *P-M* system. *P-M* dysgenesis occurs when *P* elements inherited from *P* strain males, mated with *M* strain females, cause germline cell death [67,68] due to excessive transposition in the absence of maternal *P* element piRNAs. In the *P-M* system, the asymmetry in the *P* element abundance between *P* and *M* strains can be explained by recent horizontal transfer rather than resurrection [69]. *M* laboratory strains devoid of *P* elements were established in the early part of the 20th century. *P* element invasion of natural populations via horizontal transfer occurred at a similar time, so natural populations now carry many *P* elements [70,71]. In contrast, *I-R* dysgenesis seems to have arisen from resurrected *I* elements. *I-R* dysgenesis—observed as hatch failure in eggs laid by F1 females—occurs when *I* (inducer) strain males, carrying abundant non-LTR *I* retrotransposons, mate with *R* (reactive) strain females that lack active copies [63]. However, in contrast to the P-M system, the genomes of *R* strains are littered with degraded *I* elements that are the fossils of a previous proliferation event [72,73,74,75]. In fact, under certain conditions, the degraded *I* elements can contribute to the piRNA pool and mediate repression of the newer *I* elements [76]. Thus, the genome retains a memory of past invasion and still retains some capacity to restrain new *I* elements.

Two additional cases of hybrid dysgenesis reveal a similar scenario, indicating that resurrection may be a common but poorly appreciated part of the life cycle of TEs. In *Drosophila melanogaster,* a third case of hybrid dysgenesis is driven by the *hobo* element. *hobo* is a DNA transposon that causes hybrid dysgenesis when males carrying multiple active *hobos* are mated with females that lack them [64]. Studies indicate that American populations lacked active *hobo* elements in the 1950s [77]. Strikingly, the genomes of *D. melanogaster* as well as close relatives all carry degraded copies of *hobo*. This suggests that an active version of the *hobo* element was present in an ancestor of all *D. melanogaster*, was lost, but now has proliferated to the extent that it can cause hybrid dysgenesis. This new *hobo* variant also appears among close relatives that include *D. simulans* [78,79,80,81]. Finally, a similar scenario is observed in the hybrid dysgenesis syndrome of *Drosophila virilis*. The *Penelope* element likely contributes to this syndrome and it also represents a case of re-invasion. Multiple degraded copies within the genome of *D. virilis* and relatives suggest a scenario of proliferation and extinction, followed by re-invasion [82,83].

**Figure 2:**
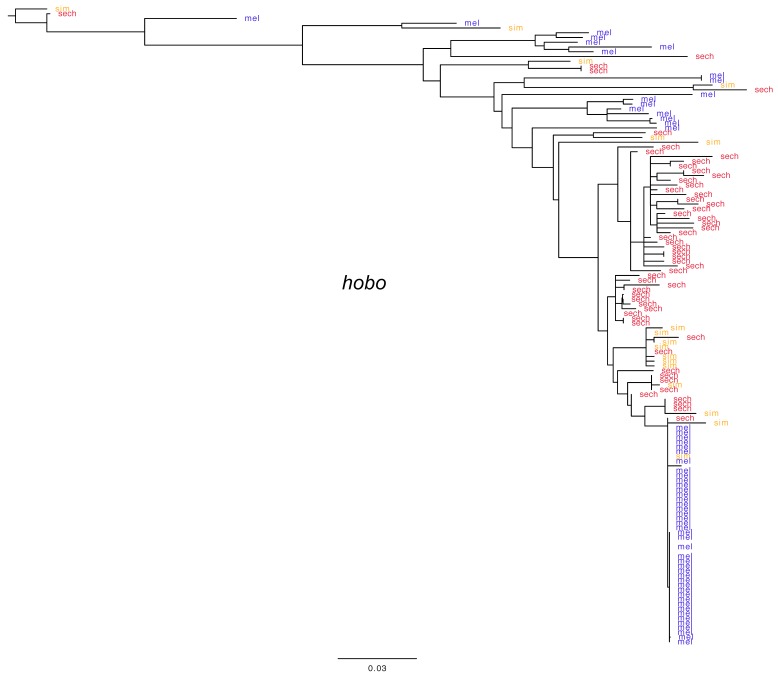
Phylogenetic analysis of *hobo* element fragments extracted from the genomes of *D. melanogaster*, *D. simulans* and *D. sechellia*. Alignments from BLAST output (E cutoff −100) were subjected to phylogenetic analysis with GARLI. Blue indicates *D. melanogaster*, red indicates *D. sechellia* and orange indicates *D. simulans*. The *hobo* phylogeny shows evidence of a previous wave in *D. melanogaster*, as well as new proliferation derived from a lineage residing within *D. simulans* or *D. sechellia*.

**Figure 3:**
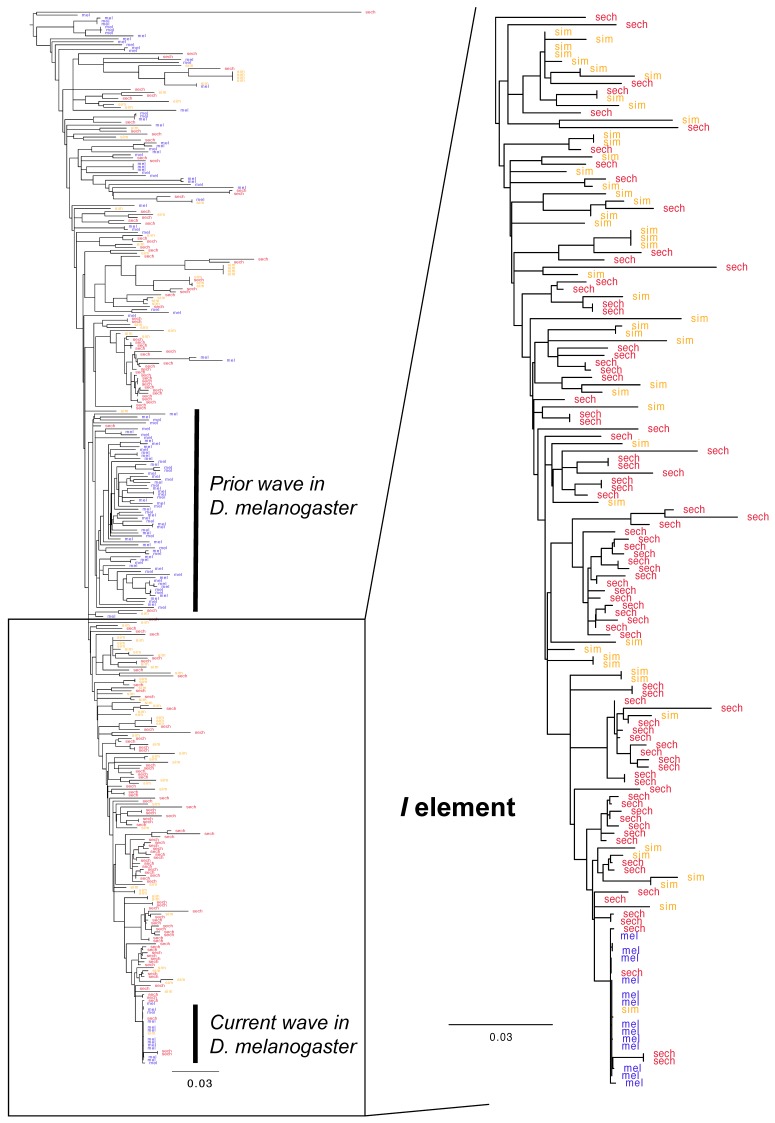
Phylogenetic analysis of *I* element fragments extracted from the genomes of *D. melanogaster*, *D. simulans* and *D. sechellia*. Alignments from BLAST output (E cutoff −100) were subjected to phylogenetic analysis with GARLI. Blue indicates *D. melanogaster*, red indicates *D. sechellia* and orange indicates *D. simulans*. A zoom-in is provided for further detail of recent dynamics. Like *hobo*, the *I* element shows evidence of a previous wave in *D. melanogaster*, as well as new proliferation derived from a lineage residing within *D. simulans* or *D. sechellia*.

As phylogenetic analysis shows (Figure 2 and Figure 3), the mode of past and current proliferation varies across these difference cases. For the *hobo* element, it appears proliferation occurred millions of years ago, prior to the divergence of *D. melanogaster* and the *D. simulans* clade. This corresponds to the upper portion of the *hobo* phylogeny where lineages of *D. melanogaster*, *D. simulans* and *D. sechellia* are intermingled in a complex manner. This was followed by another proliferation, perhaps only in the *D. simulans/D. sechellia* clade. As active and nearly identical copies currently exist in *D. melanogaster*, *D. simulans* and *D. sechellia*, a recent wave of re-activation appears to have occurred across all three species, but especially in *D. melanogaster*. Phylogenetic analysis suggests that the currently active lineage was derived from a clade of proliferating elements derived solely from *D. simulans*, as has been proposed [80]. In contrast to *hobo*, the previous proliferation of the *I* element seems more ongoing, but with one large wave of historical activity in the *D. melanogaster* genome. However, it also appears that the closest relative of the *I* element resides in *D. simulans*. This supports the possibility that both currently active variants of *hobo* and the *I* element were introduced into *D. melanogaster* from *D. simulans*. While these species do not readily produce fertile hybrids, they can in some cases.

**Figure 4:**
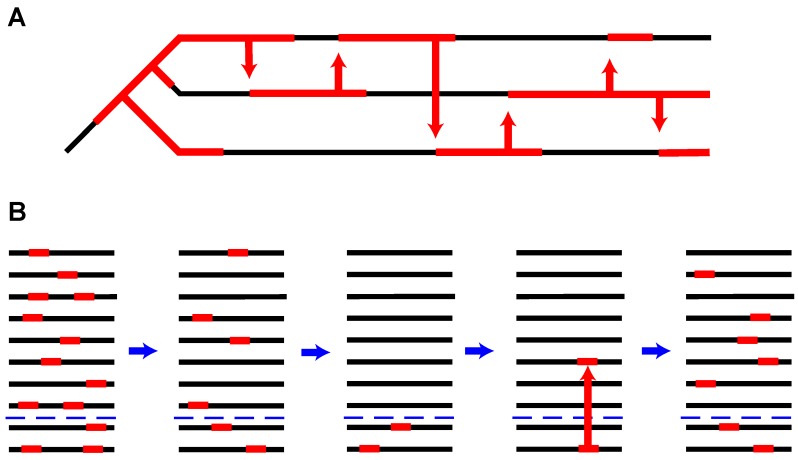
Dynamics of transposable element (TE) resurrection. (**A**) Iterated rounds of horizontal transfer (either through hybridization or other mechanisms) between sympatric close relatives allows extinction events to be followed by new rounds of proliferation. (**B**) Population subdivisions (indicated with the blue dashed line) allow a TE lineage to decrease in abundance, go extinct from one population, but become resurrected through contact with an isolated refuge population.

How does re-invasion occur? What does the time between waves of proliferation tell us about the likely mechanism of re-invasion? This is a critical question and I consider two possibilities. One possible explanation is that re-invasion arises through iterated rounds of invasion by horizontal transfer (Figure 4A). Specifically, a TE lineage residing in a reservoir species (of any kind, not just a close relative), jumps into the host. After the first round of horizontal transfer, proliferation is followed by extinction. In turn, a second horizonal transfer event is followed by an additional round of proliferation. The reservoir source species of the original horizontal transfer event may not be the same as the second. Under this mechanism, the time between proliferation events may be significant.

A second possibility is that a TE lineage becomes quiescent, but active copies linger within the species (Figure 4B). In this case, the TE lineage will have experienced only apparent, but not complete extinction. How could this occur? One scenario is that host suppression mechanisms first become sufficient so as to essentially cease transposition. This may occur through the fixation of host suppressor alleles. For example, a TE copy may land in a piRNA cluster and drive piRNA suppression that is strong enough to drive an extremely low transposition rate. Subsequently, if no TE insertion alleles are fixed within the population (due to selection against insertions), then all functional TE copies will eventually be lost by a combination of drift, selection and degeneration. However, this may take a very long period of time. During this lingering phase, several outcomes are possible. First, selection to retain host suppressor alleles (such as piRNA silencing alleles) may become reduced because there are few TE copies that remain a threat. However, if lingering TE copies take advantage of degraded suppressor alleles that have become frequent within the population, the TE lineage may return to an active state and in turn prevent the fixation of degraded host suppressor alleles. Depending on the timing of these dynamics, this may simply appear to represent a case of continued proliferation. An alternate outcome may give the illusion of resurrection. In this case, host suppression alleles may become degraded and these degraded variants may increase in frequency and perhaps even fix. In the scenario where non-functional host suppressor alleles are neutral, perhaps due to an exceedingly low abundance of lingering TE copies, the time until suppressor decay will be similar to the rate of mutation to the non-functional state. This may be of the order of millions of generations and it may be unlikely that an active lineage could persist for this time. But if lingering TE copies persist, the loss of a host suppressor allele may lead to a new round of TE proliferation. This scenario may be more likely if isolated populations can function as source refugia for new waves of TE proliferation. Depending on the timing of these dynamics, this may give the appearance of TE resurrection. Even if a host suppression allele is not lost, TE resurrection may also be apparent if a novel TE variant arises during the lingering phase. Such a variant may confer resistance to the host suppression allele, thus allowing a new round of TE proliferation. This would be analogous to evolutionary rescue of the TE lineage. Finally, true resurrection may be possible if recombination between non-functional copies leads to restoration of a functional copy.

These two scenarios may explain the appearance of Lazarus elements. In the first case, iterated rounds of horizontal transfer give the appearance of resurrection. In the latter case, an active lineage becomes quiescent for a duration, but lingers. After some period of time, it becomes active again. The key distinction between these two scenarios is the source for the newly proliferating lineage. Is it “from without” or “from within”? The likelihood of these two explanations depends on a large number of unknown parameters. It is apparent that horizontal transfer is quite common for transposable elements and this fact lends support to the “from without” hypothesis. In fact, at least for *Drosophila*, it appears that iterated rounds of horizontal transfer among close relatives might play an important role in the dynamics of re-invasion. For both the *hobo* and the *I* element, copies shared among close relatives are highly similar. Thus, rare hybridization may contribute to continued TE exchange and enable iterated bouts of re-invasion. Horizontal transfer of TEs among close relatives of *D. melanogaster* appears rampant [84]. A similar pattern of ongoing exchange of TEs also appears in close relatives of *D. pseudoobscura* that are in sympatry [85]. Overall, the genomes of closely related species in sympatry may function as an effective higher-level ecosystem for TEs. As modes of TE silencing in one species decay, horizontal transfer mediated by either hybridization or other mechanisms may allow iterated rounds of re-introduction. This dynamic is likely to be shaped by the dynamics of decay of host suppressor alleles. Nonetheless, it is also apparent that systems of host suppression are in rapid flux, and selection for host suppression in any single species may be quite weak. Thus, host alleles that suppress TE movement—such as TE insertions into piRNA clusters—may decay after the threat imposed by a TE family becomes reduced, even if the TE lineage has not yet become completely extinct within the species. In this case, new proliferation events may arise “from within”. How might we test between these hypotheses? The key may be to identify the source refugia for resurrected TEs. For the “from without” hypothesis, these may be other species that live in physical proximity, such as close relatives that can hybridize. Alternately, shared parasites that enable horizontal transfer may also function as refugia [86]. However, horizontal transfer can be a very rare event, so proof of source may be extremely challenging. For the “from within” hypothesis, this may require a closer study of TE dynamics in large populations that have geographic structure. Theoretical studies may also examine whether it is plausible for a functional TE lineage to persist at low frequency until a host suppressor allele is lost, followed by re-invasion. Examples of apparent resurrection, where divergence between active and degraded copies are in the 5% to 10% range, suggest that the time would perhaps be too long. However, in the case of the *I* element where degraded copies may be quite young, the “from within” hypothesis might be more plausible. Altogether, distinguishing between these two possibilities will provide insight into the evolutionary strategies that TEs employ to ensure their continued presence across diverse species.
